# Two Terpene Synthases Are Involved in Multiple Sesquiterpene Biosynthesis in the Woody Vegetable, *Toona sinensis*

**DOI:** 10.3390/ijms26041578

**Published:** 2025-02-13

**Authors:** Yaping Zheng, Wenjing Li, Jianhua Dai, Yaoyi Zhang, Minyan Wang, Jun Liu, Hengfu Yin, Haimei Li

**Affiliations:** 1 College of Landscape Architecture and Forestry, Qingdao Agriculture University, Qingdao 266000, China; 15658139523@163.com; 2 Research Institute of Subtropical Forestry, Chinese Academy of Forestry, Hangzhou 311400, China; liwenjing0217@163.com (W.L.); gianfadai@163.com (J.D.); 15191249657@163.com (Y.Z.); w524270986@163.com (M.W.); ywliu2005@163.com (J.L.)

**Keywords:** glandular trichome, sesquiterpenes, *Terpene Synthase*, *Toona sinensis*, secondary metabolism

## Abstract

As a special woody vegetable, Chinese toon (*Toona sinensis*) has a unique flavor, which is mainly formed by a combination of volatile substances. The secretion and storage of volatile odorants in plants are often carried out in trichomes. Currently, studies on the formation of *T. sinensis* flavor in terms of biosynthetic processes and epidermal trichome morphology are scarce. Here, we conducted a detailed analysis of the morphology, structure, and distribution of trichomes on the leaves of *T. sinensis*. We identified three types of trichomes: non-glandular, sessile glandular, and stalked glandular. We found that the distribution of trichomes varies greatly in the natural populations of *T. sinensis*, and this may be closely related to the changes in volatile components. In order to clarify the relationship between secondary metabolism and trichome formation, we integrated the metabolic analysis of volatiles with transcriptome analysis and discovered two important (*Terpene Synthase*) *TPS* genes that may be directly involved in terpene synthesis. Through the heterologous expression in tobacco and the transient expression in *T. sinensis*, we showed that the *TPS* genes can participate in the synthesis of sesquiterpenes, among which *TsTPS1262* can lead to the synthesis of elemene in *T. sinensis*. Our study provides insights into the synthesis pathways of complex volatile components in *T. sinensis* and also provides a basis for flavor breeding applications.

## 1. Introduction

*Toona sinensis*, a species of the Meliaceae family *Toona* genus, is widely distributed in East and Southeast Asia [1]. *T. sinensis* is domesticated as an important woody vegetable in the traditional Chinese diet, largely due to the unique flavor and high nutritional value in its young shoots and leaves [2]. The domestication of *T. sinensis* can be traced back to more than 2000 years ago, and in the Tang Dynasty, “Heiyouchun” was an imperial tribute [3,4]. In addition to being a tasty food, *T. sinensis* has great medical benefits for people. Its leaves contain a large number of bioactive compounds, including terpenoids, flavonoids, sterols, and saponins, which have significant antitumor, antioxidant, and hepatic fibrosis-relieving effects [1,5].

*T. sinensis* is best known for its distinctive aroma. The flavor of *T. sinensis* is characterized by an onion-like feature, which is mainly attributed to its volatile substance composition [6,7,8,9]. Studies have shown that the volatile components of *T. sinensis* contain unique sulfur-containing substances (VSCs), terpenoids, and others, and the integration of VSC and terpenoids is thought to cause the unique aroma of *T. sinensis* [8,9,10]. The VSCs of *T. sinensis* have been found to be predominantly thiophenes such as 2-mercapto-3,4-dimethyl-2,3-dihydrothiophene. Although the biosynthetic pathway of thiophene has not been completely clarified, it has been reported that the biosynthetic precursor of thiophene is possibly oleic acid [11,12]. Terpenoids, members of the major volatile organic compounds (VOCs) associated with the formation of the distinctive odor of *T. sinensis*, are a class of structurally diverse natural compounds [13]. Currently, the biosynthetic pathways of terpenoids are well defined. Based on the Carbon number of the compound, terpenes can be categorized into four groups, sesquiterpenes (C5), monoterpenes (C10), sesquiterpenes (C15), and diterpenes (C20) [14]. Depending on the terpene product, the main biosynthetic processes can be divided into the mevalonate (MVA) pathway and the 2-C-methyl-4-erythritol phosphate (MEP) pathway, of which, in most cases, monoterpenes/diterpenes go through the MEP pathway, and sesquiterpenes go through the MVA pathway [15,16]. In recent years, gas chromatography–mass spectrometry (GC-MS) analysis methods have been used to study the components of volatile substances in *T. sinensis* [3,13,17,18,19]. Although the composition of the components in different samples has obvious differences, in general, the volatile substances are composed of VSC and terpenes [20,21,22]. In particular, a variety of sesquiterpene components were found to account for the majority of terpene components in different *T. sinensis* varieties or tissues, such as caryophyllene, copaene, elemene, and other components [23,24,25,26,27]. However, little is known about the biosynthetic pathways of the volatile terpenoid components of *T. sinensis*.

Plant trichomes are usually tissues for the biosynthesis of secondary metabolites. In general, plant trichomes can be divided into two major categories based on their morphology and secretion capacity: glandular trichomes and non-glandular trichomes [16,28]. The morphology of glandular trichomes in different plants is diverse. They are typically multicellular; composed of differentiated basal, stalk, and apical cells; and produce a large number of different classes of specialized metabolites [29,30,31]. The morphological changes in glandular trichomes appear to be related to adaptation to the types of compounds they produce [32]. In tomatoes, there are eight types of trichomes, including glandular trichomes (types I, IV, VI, and VII) and non-glandular trichomes (types II, III, V, and VIII) [33], which are involved in the synthesis, storage, and secretion of various specialized metabolites such as terpenoids, phenylpropanoids, flavonoids, alkaloids, and acyl sugars [30]. For example, the terpene synthase gene *Phellandrene Synthase 1* (*PHS1*) is specifically expressed in glandular trichomes and is able to catalyze the synthesis of phellandrene as the main product and various other monoterpenes [34].

The *TPS* gene family has an important role in terpene biosynthesis. In *Arabidopsis thaliana* and tomato, which are representative plants, the TPS gene family can be divided into seven main branches: *TPS*-a to *TPS*-h, among which TPS-a mainly contains sesquiterpene and diterpene synthases, and TPS-b and TPS-g branch mainly consists of monoterpene synthases [35,36]. Based on the protein structural characteristics, TPS can specifically catalyze the intermediates involved in the terpene biosynthesis pathway, such as dimethylallyl diphosphate (DMAPP and C5), geranyl diphosphate (GPP and C10), farnesyl diphosphate (FPP and C15), and geranylgeranyl diphosphate (GGPP and C20), thereby forming C5, C10, C15, and C20 terpene skeletons [23]. Taking sesquiterpenes as an example, the starting substrate of TPS is farnesyl diphosphate (FPP), and most of its TPS conserved motifs contain “DDXXD/E” and “DTE/NSE” [37]. For example, *AtTPS11* and *AtTPS21* are involved in catalyzing the synthesis of various sesquiterpenes in flower volatiles of *Arabidopsis thaliana* [23]. In *T. sinensis*, *TsTPS18* was found to be involved in the regulation of caryophyllene synthesis [17]. Although the *TPS* gene is of great significance in improving the flavor, currently, our understanding of TPS in *T. sinensis* is still very limited.

In this study, we characterized the distribution density changes and morphological structure of epidermal trichomes in the natural populations of *T. sinensis* and performed a global gene expression analysis to identify the key genes that are associated with trichome development and secondary metabolic pathways. Combining gene expression and trichome distribution analysis, we identified and characterized two putative sesquiterpene synthase genes, *TsTPS466* and *TsTPS1262*. Through heterologous transformation in model plants and *T. sinensis* and compositional analysis of VOC components, we showed that these two TPS genes are important genes involved in sesquiterpene biosynthesis. Our research results provide a molecular basis for elucidating the formation of *T. sinensis* flavor traits and provide basic information for the breeding of high-quality varieties.

## 2. Results

### 2.1. T. sinensis Varieties from Different Geographical Regions Have Significant Differences in Trichome Density

**Figure 1:**
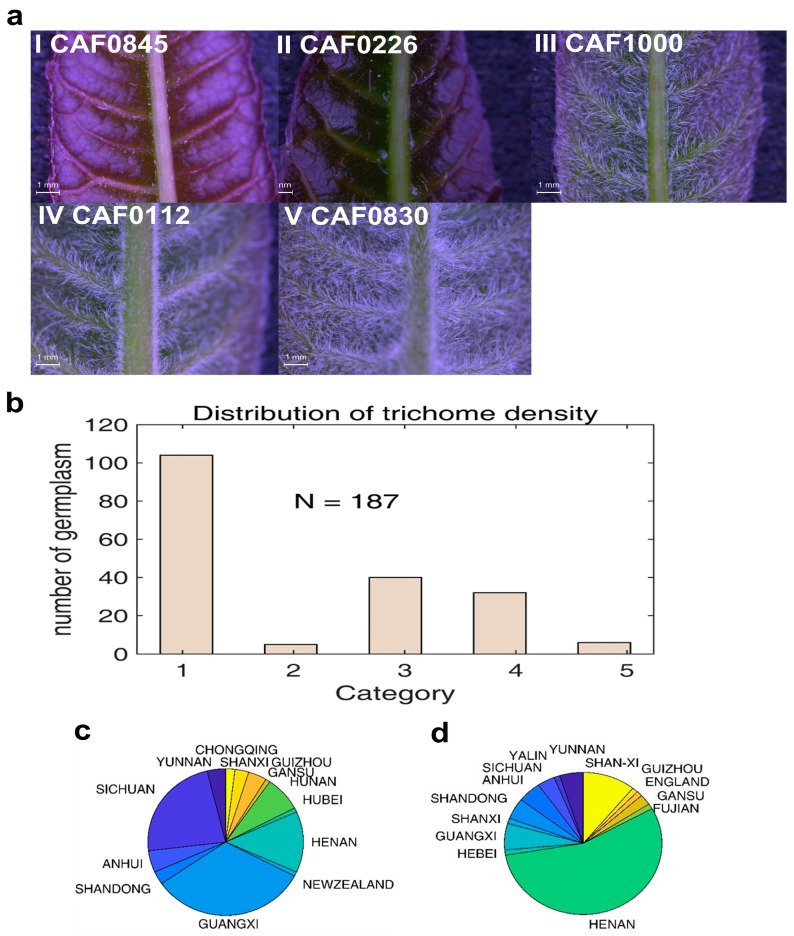
Variation in trichome density in *T. sinensis* and distribution pattern of geographical origins. (**a**) Based on the variation in trichome density on the leaves of *T. sinensis*, the breeding populations were grouped into five types. Representative leaves (abaxial surface) were visualized using light-dissection microscopy. (**b**) Distribution of different categories of germplasms using the breeding population of 187 individuals. (**c**) Geographical origin of the category I varieties. (**d**) Geographical origin of the category II–V varieties.

As a specialized plant structure, trichomes play an important role in adaptive evolution by acting as a protective shield against temperature extremes, ultraviolet radiation, and insects [38]. We found that the distribution density of trichomes varied significantly among *T. sinensis* species originating from different regions; we roughly categorized them into five types based on the density gradient and selected representative species for each of them (Figure 1a). In order to clarify the origin of each type, we analyzed 187 *T. sinensis* varieties and found that among them, most of the varieties with low trichome density belonged to type-I (Figure 1b), which were mainly distributed in the southern regions of China such as the Guangxi Autonomous Region and Sichuan province (Figure 1c). We found that the II-type to V-type varieties with a relatively high density of epidermal trichomes were mainly distributed in the northern regions of China, such as Henan, Shanxi, Shandong, etc.; for example, a representative variety CAF0830 with high trichome density was collected from Henan province (Figure 1d). Overall, the density of trichomes varies geographically between the north and south regions of China, possibly resulting from the evolution of environmental adaptations.

### 2.2. Morphological Characterization of Glandular and Non-Glandular Trichomes of T. sinensis

**Figure 2:**
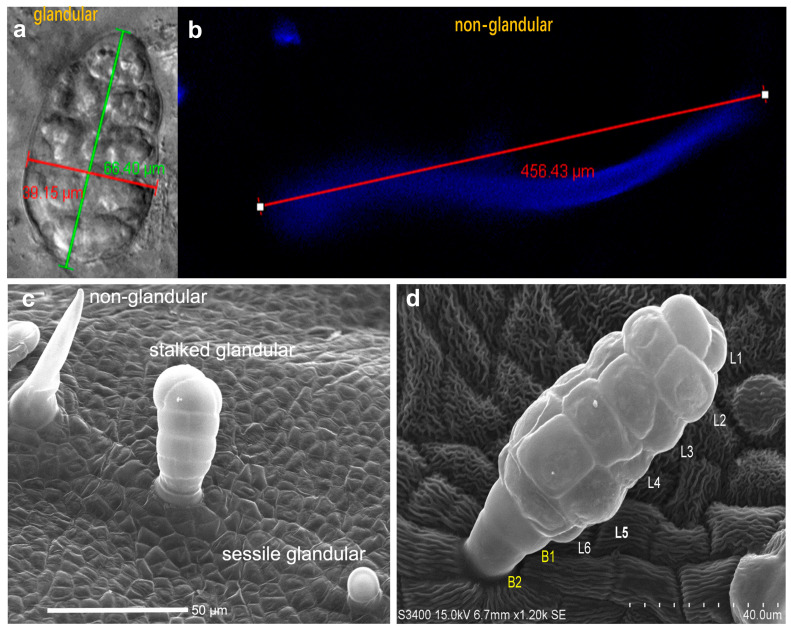
Morphological analysis of different types of trichomes in *T. sinensis*. (**a**) Laser microscopy image of a glandular trichome. (**b**) Laser microscopy image of a non-glandular trichome that has autofluorescence under UV conditions. (**c**) Scanning electron microscopy image of *T. sinensis* leaf surface, containing stalked glandular, sessile glandular, and non-glandular trichomes. (**d**) Morphological analysis of a staled glandular trichome under scanning electron microscopy. Two basal cells (B1 and B2), and six layers of cells are indicated by L1 to L6.

To further understand the morphological structure of trichomes, we studied the trichomes of *T. sinensis* leaves using scanning electron microscopy and laser confocal microscopy. By real-time imaging confocal laser microscopy, we observed two unique trichomes: one is a typical hair-like structure with UV-excited autofluorescence and is non-glandular; the other is an epidermal trichome with weak autofluorescence signal at the base (Figure 2b), and this trichome is composed of multiple cells and has an oval shape (Figure 2a). To further characterize the morphology of trichomes, we performed an SEM analysis. We found that *T. sinensis* has at least three types of trichomes: sessile glandular, stalked glandular, and non-glandular trichomes, which differ greatly in morphology, structure, and cellular composition (Figure 2c). We found that both the non-glandular trichomes and glandular trichomes of *T. sinensis* were attached to the leaf epidermis, which might have developed directly from leaf epidermal cells. The non-glandular trichomes were typical unicellular trichomes, which were needle-like and had no basal cells (Figure 2b,c), and the length of the developed mature non-glandular trichomes observed by laser microscopy could reach 456 μm (Figure 2b). The stalked glandular trichomes were generally elliptical in shape (Figure 2a,c) and had a multicellular tower-like arrangement morphology with 2–3 basal cells at the base and 6 layers of cells arranged to form the head at the top (Figure 2a,d). Sessile glandular trichomes are shorter than stalked glandular trichomes, may be composed of single cells, and have a significant head space area, indicating that they may be storage sites for volatiles (Figure 2c). Compared with the non-glandular trichomes, the glandular trichomes were shorter, with distinct nuclei and other organelles in the apical and middle cells, suggesting that they may have an active function in the synthesis of secondary metabolites.

### 2.3. Changes in the Distribution of Epidermal Trichomes and Differences in Their Volatile Substances Among Varieties of T. sinensis

**Figure 3:**
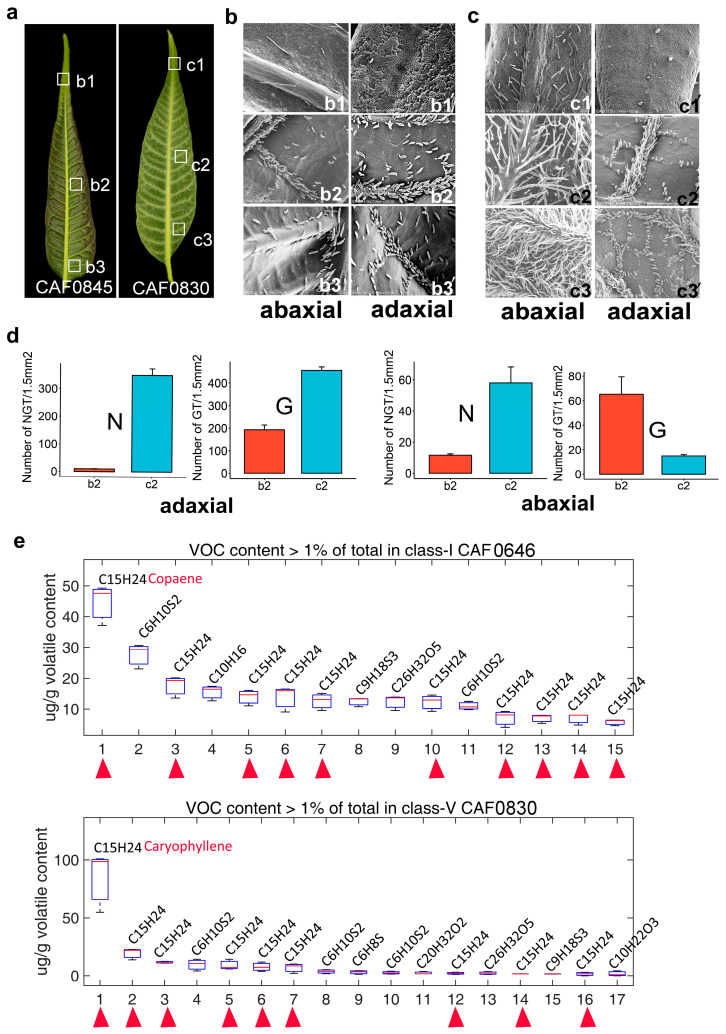
Characteristics of the distribution of epidermal trichomes and the analysis of composition changes in volatile substances. (**a**) The tip, middle, and base regions of the young leaves of the representative varieties (CAF0845 and CAF0830) (shown in white boxes) were selected for the phenotypic analysis of the distribution of epidermal trichomes. The SEM analysis of the upper (adaxial) and lower (abaxial) surfaces of the leaf tip, middle, and base of the CAF0845 (**b**) and CAF0830 (**c**) leaves. (**b1**–**b3**), abaxial side of leaf surface from the tip, middle and base of CAF0845 respectively; (**b1’**–**b3’**), abaxial side of leaf surface from the tip, middle, and base of CAF0845 respectively; (**c1**–**c3**), abaxial side of leaf surface from the tip, middle and base of CAF0830 respectively; (**c1’**–**c3’**), abaxial side of leaf surface from the tip, middle, and base of CAF0830 respectively. (**d**) Statistical analysis of the glandular and non-glandular trichomes on the adaxial and abaxial surfaces of the mid-section of the leaves of CAF0845 and CAF0830, in which the number of epidermal trichomes within an area of 1.5 mm^2^ was counted. N, non-glandular; G, glandular. (**e**) The volatile components in the leaves of CAF0646 and CAF0830 were measured by GC-MS, and the components accounting for more than 1% of the total content were listed. The components corresponding to the chemical formulas are shown in Table 1. The red arrow shows the sesquiterpene component.

To investigate the relationship between epidermal trichomes and volatile components, we examined two typical varieties, CAF0845 (type-I) and CAF0830 (type-V), with significant differences in the epidermal trichome density (Figure 3a). We performed SEM analyses on three representative regions (apical, middle, and basal) of the adaxial and abaxial surfaces of young leaves in each variety, respectively. We observed that the number of trichomes in the apical region was the lowest in both species (Figure 3b,c), and only a small number of predominantly non-glandular trichomes were observed at the dorsal tip of CAF0830 (Figure 3c). Based on the SEM analysis, the CAF0830 variety has more trichomes in all the corresponding regions than the CAF0845 variety (Figure 3b,c); however, both varieties have a large number of glandular and non-glandular trichomes in the central region, especially the distal axial surface which is predominantly glandular trichomes (Figure 3c). To statistically analyze the differences in trichomes between the two varieties, we compared the number of glandular and non-glandular trichomes on the adaxial and abaxial surfaces of the middle region of leaves. We showed that the number of glandular trichomes was higher in CAF0845 than in CAF0830 in the adaxial surface (Figure 3d), and the number of glandular and non-glandular trichomes was higher in CAF0830 than in CAF0845 in all the other regions (Figure 3d).

To analyze the relationship between volatiles and trichomes, we measured the volatiles of the leaves of two representative varieties (Table 1, type-I CAF0646 and type-V CAF0830). We found that although the composition and content of the constituents varied considerably, the main volatiles consisted of sulfur-containing thiophenes and sesquiterpenes (Figure 3e). In the type-I variety, we detected 10 sesquiterpenes, with copaene being the most abundant (Figure 3e); in the type-V variety, we 9 sesquiterpenes, of which caryophyllene was the most abundant component (Figure 3e). By comparison, we found that the differences in the compositions and contents of sesquiterpenes in type-I and type-V varieties were notable, especially in type-V, where the caryophyllene fraction accounted for 43% of the total volatiles, but in type-I, the sesquiterpene compositions were more complex and contained a monoterpene component (Figure 3e).

### 2.4. Comparative Transcriptomics Analysis Reveals Genes Involved in Secondary Metabolism Are Associated with Trichome Density

In order to study the expression of the genes related to trichome development, we performed an RNA-Seq analysis on different regions of the leaves of the two *T. sinensis* varieties. In total, we obtained the sequencing results for 18 transcriptome samples with an average number of valid reads of 11.69Gb (Table S1). By alignment with the reference genome of *T. sinensis*, we obtained the expression levels of the genes. We performed a correlation analysis of the gene expression of the samples and showed that the replications of the samples were highly correlated with each other (Figure S1), suggesting that the transcriptomic data are reliable and suitable for statistical analysis.

**Figure 4:**
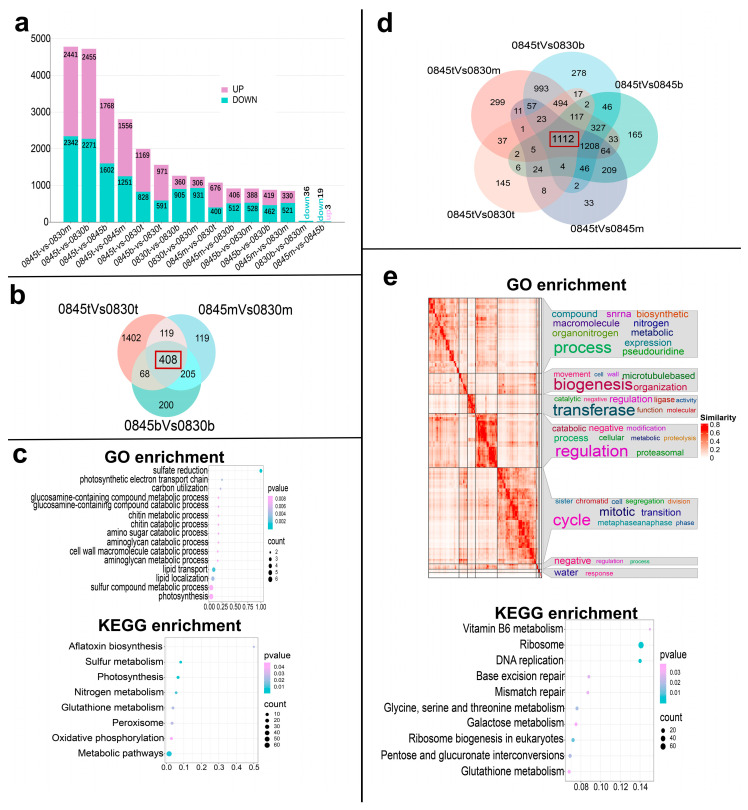
Comparative transcriptome analysis of the different leaf zones in the *T. sinensis* varieties with differential trichome density. (**a**) Summary of the distribution of the DEGs among comparisons of the samples. (**b**) A Venn diagram illustrating the DEGs at the same site across two genotypes. (**c**) GO function enrichment (*p*-value < 0.01) and KEGG pathway enrichment (*p*-value < 0.05) results from the bubble plots for the common genes in Figure 4b. (**d**) A Venn diagram depicting the top five groups with the highest number of DEGs. (**e**) GO function enrichment (*p*-value < 0.01) and KEGG pathway enrichment (*p*-value < 0.05) plots for the common genes in Figure 4d.

We further performed gene expression analysis to obtain the differentially expressed genes (DEGs) (Log (FC) ≥ 1 and FDR ≤ 0.05) for several comparisons. The results showed that in different comparison combinations, the number of DEGs in the top and middle regions between the two varieties was high, while the number of DEGs in the middle and bottom regions of the same variety was significantly less (Figure 4a); moreover, it can be found that in the combinations between two genotypes, the number of DEGs was significantly higher (Figure 4a). In order to explore the distribution of DEGs, we conducted multiple combination analyses. First, we identified the DEGs that were compared at the same locations on the CAF0845 and CAF0830 leaves and identified 408 shared differential genes (Figure 4b). GO and KEGG enrichment analysis of the shared DEGs indicated that secondary metabolic pathways, including sulfur-containing substances, lipids, and amino acid metabolism, were involved in the differential process (Figure 4c). Second, we selected five combinations with a high number of DEGs for analysis, from which we identified 1112 shared DEGs (Figure 4d). The following GO and KEGG enrichment analyses of these 1112 DEGs revealed that similar to previous analyses, multiple secondary metabolic pathways are also the main pathways involved in these differential genes (Figure 4e). Taking these results, we suggest that the variations in trichome density in *T. sinensis* are closely related to the process of the biosynthesis and regulation of secondary metabolites and may share a common regulatory pathway.

### 2.5. Characterization of TPS Genes in T. sinensis Identify Candidates Involved in Sesquiterpenoid Biosynthesis

**Figure 5:**
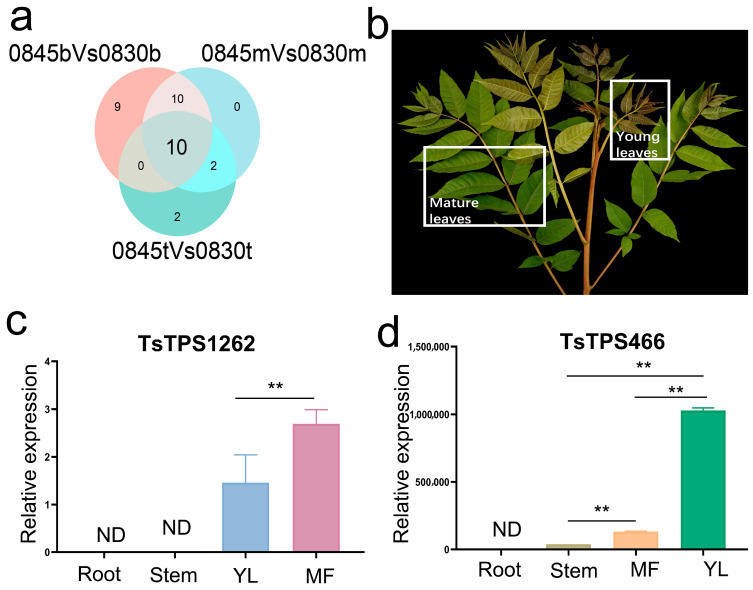
Identification of candidate TPS genes in *T. sinensis*. (**a**) A Venn diagram illustrating the differentially expressed TPS genes at the same leaf position across two varieties, with *TsTPS1262* and *TsTPS466* located among the intersecting 10 genes (**b**) Sampling of tissues for gene expression validation. The white boxes show the sampling sites of the mature and young leaves of *T. sinensis*. (**c**,**d**) RT-PCR analysis of *TsTPS1262* and *TsTPS466* in the roots, stems, mature leaves, and young leaves of *T. sinensis*. ** indicates significant changes by *p*-value < 0.01. ND, not detected.

We focused on the variation in the sesquiterpene content in the T. sinensis varieties and analyzed the expression levels of the TPS gene family (Table S2). In total, we obtained 44 *TPS* genes in *T. sinensis* based on our transcriptome data, and their expression patterns varied among varieties and regions (Figure S2). We analyzed the expression levels of TPS genes differentially among the varieties and identified 10 TPS genes that were differentially expressed in all three corresponding regions of the two varieties (Figure 5a). Among them, we identified two genes (*TsTPS1262* and *TsTPS466*) that are highly similar to the *AtTPS21* gene involved in sesquiterpene synthesis in Arabidopsis [23]. We further analyzed the expression profiles of *TsTPS1262* and *TsTPS466* in different tissue types of *T. sinensis* (Figure 5b). We found that both genes were highly expressed in the leaves, *TsTPS1262* mainly in the mature leaves and *TsTPS466* in the young leaves (Figure 5c,d).

**Figure 6:**
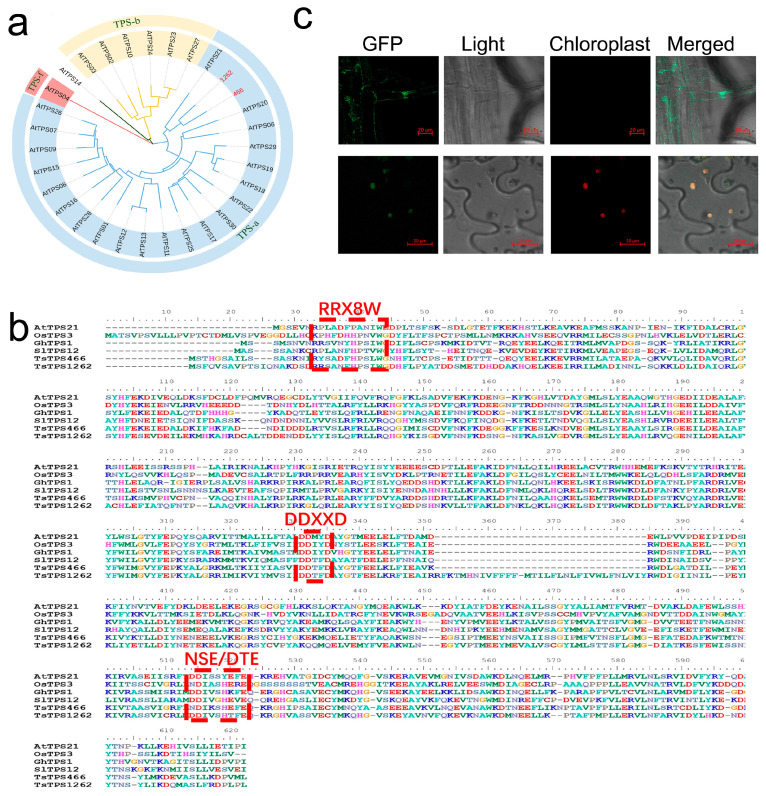
Phylogenetic analysis and subcellular localization of TPS genes from *T. sinensis*. (**a**) The phylogenetic analysis of the TPS genes from *Arabidopsis thaliana* and *T. sinensis*. (**b**) A multiple sequence comparison of terpene synthase (TPS) genes from *T. sinensis*, *Arabidopsis thaliana*, *Solanum lycopersicum*, *Oryza sativa*, and *Gossypium hirsutum* was conducted. The red rectangles indicate the conserved structural domains. (**c**) Subcellular localization analysis of *TsTPS1262* was conducted using leaf and root tip tissues from a positive line of transgenic *Arabidopsis thaliana*.

We cloned and verified the full coding sequences of *TsTPS1262* and *TsTPS466* and performed a phylogenetic analysis of the TPS genes from *Arabidopsis*. Both *TsTPS1262* and *TsTPS466* were closely related to *AtTPS21* (Figure 6a), suggesting functional relevance in sesquiterpenoid biosynthesis. Further, through multiple sequences alignment, we showed that *TsTPS1262* and *TsTPS466* have a high degree of sequence similarity with *AtTPS21*, including “DDXXD”, “RRX8W”, and “NST/DTE” signatures in conserved catalytic motifs (Figure 6b). We constructed a *TsTPS1262*-GFP fusion expression vector to study cellular localization. By transforming *Arabidopsis thaliana*, we obtained a stable positive transgenic line. Through confocal observation, we found that the *TsTPS1262*-GFP signal was localized in chloroplasts in leaves, while the observation of root cells revealed that it was localized in the cytoplasm (Figure 6c).

### 2.6. Ectopic Expression of TsTPS1262 and TsTPS466 Leads to Complex Sesquiterpene Biosynthesis in Tobacco and T. sinensis

**Figure 7:**
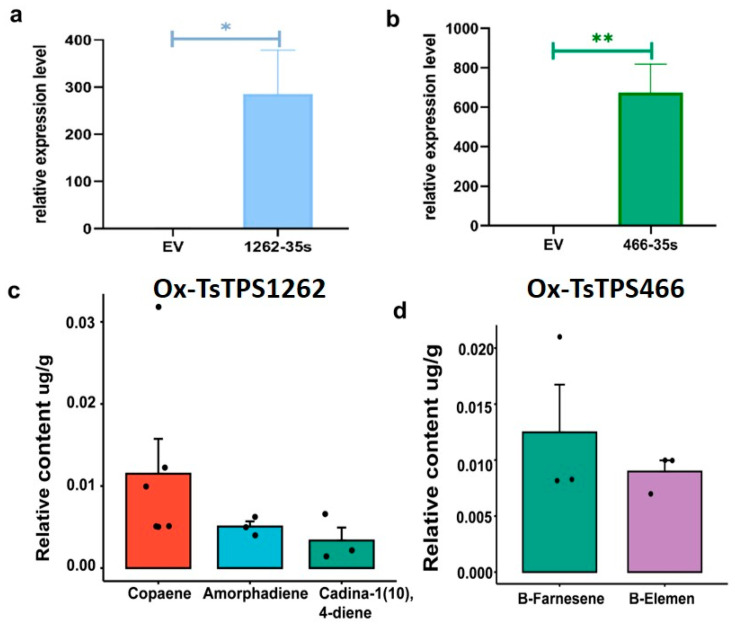
Ectopic expression of *TsTPS1262* and *TsTPS466* in tobacco and the analysis of volatiles. Relative gene expression in tobacco leaves following the ectopic expression of *TsTPS1262* (**a**) and *TsTPS466* (**b**). * indicate significant changes by *p*-value < 0.05 and ** indicates significant changes by *p*-value < 0.01. The identification of newly formed sesquiterpenes in the ectopic expression of *TsTPS1262* (**c**) and *TsTPS466* (**d**) in the leaves of tobacco compared to empty vector (EV). For transient expression, each experiment consisted of six replicates of the volatility analysis, and only sesquiterpene components identified at least three times were shown. Each point in (**c**,**d**) represents the results of an independent experiment.

To investigate the function of secondary metabolism, we ectopically expressed the TsTPS1262 and TsTPS466 genes in tobacco. We showed that transient expression can effectively induce a high expression of candidate genes (Figure 7a,b). Based on this, we measured the content and composition of volatiles in transgenic leaves. Both the overexpression of TsTPS1262 and TsTPS466 resulted in a variety of terpene profiles (Table S3). We found that a variety of sesquiterpenes were present in the overexpression plants but not in the control (Figure 7c,d). Specifically, in the overexpression of TsTPS1262, we identified three sesquiterpene constituents; in the overexpression of TsTPS466, we identified two sesquiterpene constituents (Figure 7c,d).

**Figure 8:**
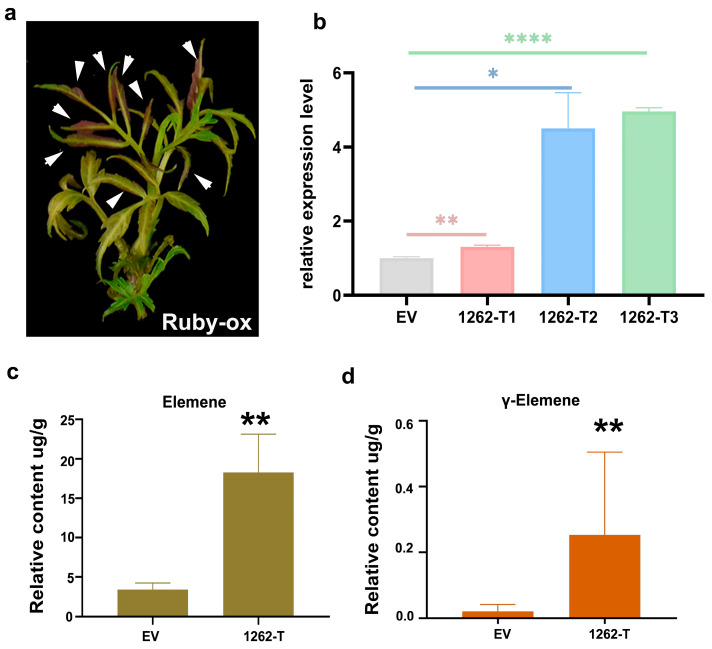
Establishment of a transient expression system in *T. sinensis* seedlings and the detection of volatile substances. (**a**) The high efficiency of the transient expression system in *T. sinensis* was demonstrated by the pigmentation of the RUBY reporter vector. (**b**) Relative expression of *TsTPS1262* in transgenic *T. sinensis* leaves. (**c**) The content of sesquiterpene elemene was highly induced in transgenic materials of *T. sinensis* compared to empty vector control. Three biological replicates are used for statistical analysis. (**d**) The second abundant sesquiterpene γ-elemene was significantly induced. Three biological replicates are used for statistical analysis. * indicates significant changes by *p*-value < 0.05, ** indicates significant changes by *p*-value < 0.01, and **** indicates significant changes by *p*-value < 0.0001.

In order to further study the biological function, we established an overexpression system in *T. sinensis* based on vacuum filtration. First, we tested the transformation efficiency through the RUBY reporter gene and found that more than half of the young leaves of a single transformant had a red signal expression, indicating that the overexpression was effective (Figure 8a). We analyzed the overexpression of *TsTPS1262* and found that *TsTPS1262* gene expression was up-regulated in three independent transformations, and two transformants were significantly up-regulated (Figure 8b). We detected the volatile components of the transformants by GC-MS (Table S4) and found that the sesquiterpene components of elemene and γ-elemene were significantly detected in three replicates (Figure 8c,d).

## 3. Discussion

Our findings on the molecular characterization of trichome formation and volatile biosynthesis in *T. sinensis* are consistent with previous studies in other plants. For instance, similar TPSs have been identified in *Arabidopsis thaliana* and *Solanum lycopersicum*, where they play crucial roles in sesquiterpene biosynthesis [23,36,39]. However, unlike these species, the GC-MS analysis showed that *T. sinensis* exhibits unique volatile compound profiles, which may be attributed to the production of specific volatile compounds (such as VSC in Table 1) components; their formation may be the product of the adaptive evolution. Comparative studies of the *TPS* gene family using a variety of angiosperms have shown that the expansion and functional differences in TPS contribute to the adaptability and diversity of angiosperms and plants, promoting the production of broad-spectrum terpenes [40]. Our study not only enhances our understanding of volatile biosynthesis but also provides a potential target for improving the production of valuable compounds in woody vegetables.

*T. sinensis* is widely distributed in China and is an edible vegetable due to its unique flavor. Research has found that it is rich in a variety of compounds with medicinal value, and has been found to contain more than 200 compounds, such as triterpenes, sesquiterpenes, diterpenes, sterols, phenols, flavonoids, phenylpropanoids, and other ingredients [41]. The volatile substances of *T. sinensis* varieties originating from the three regions of Shandong Province, Henan Province, and Anhui Province in China were analyzed by GC-MS, and it was found that the main components are terpenes, thiophenes, and esters [9]. This is similar to our results of measurements using varieties with different trichome densities: both found sesquiterpenes to be the main terpene component (Figure 3e). However, we found that the volatile compounds in the *T. sinensis* leaves have significant differences among varieties (Figure 3e), suggesting that the synthesis of VOCs is closely related to trichome development.

The formation and variation in *T. sinensis* flavor are the basis of artificial selection, in which the component changes in VOCs are regulated by the synthesis pathway of secondary metabolites. The GC-MS analysis of different varieties of *T. sinensis* shows that the content of terpenoids (especially sesquiterpenes) and VSCs in VOCs are the main component substances [42]. Studies on the enzyme activities of five sesquiterpene synthases in grapes show that there are significant differences in the final products catalyzed by different TPSs, which indicates the complexity of sesquiterpene components and diverse functions of TPS genes [43]. At present, some progress has been made in the research on the synthesis and regulation of terpenoids in *T. sinensis*. For example, two key terpene synthase genes, *TsTPS1* and *TsTPS2*, were identified in *T. sinensis* and found to be specifically expressed in trichomes and regulate the in vitro biosynthesis of (+) limonene and β-elemene, respectively [44]. In addition, *TsTPS18* was found to be a sesquiterpene synthase, and its ectopic expression in *T. sinensis* has been shown to promote the synthesis of β-caryophyllene [17]. Based on the sequence characteristics and metabolite correlation analysis, this study preliminarily identified *TsTPS1262* and *TsTPS466* in *T. sinensis* as sesquiterpene synthases (Figure 5). We used transient expression systems in tobacco and *T. sinensis* to study the function of catalyzing the production of sesquiterpenes. Our transient expression analysis showed increased copaene and elemene contents (Figure 7 and Figure 8), which is similar to results previously reported in *Arabidopsis* [23]. We found that the composition of sesquiterpenes in tobacco and *T. sinensis* transient expression systems was different (Figure 7 and Figure 8). This may be related to the differences in precursor substances in the recipient plants, which also indicates that the TPS genes may have broad activity on producing sesquiterpenes.

Sesquiterpenes are mainly synthesized from the precursor FPP in the cytoplasm through the MVA pathway, and sesquiterpene synthases are also usually localized in the cytoplasm [13,23,45]. The current research suggests that precursors for terpene synthesis may be transported within cells, complicating the synthesis pathway [46]. For example, the tobacco sesquiterpene synthase *NtTPS21* has also been shown to be localized in the nucleus [47]. Our results showed that transgenic *TsTPS1262*-GFP fusion protein is located in the cytoplasm of Arabidopsis root cells, while in leaves, *TsTPS1262*-GFP is located in the chloroplasts (Figure 6). To clarify its functional pathway, more experimental evidence and further clarification and discussion of subcellular localization are needed.

Trichomes are widely distributed on the surface of many plants, and their functions vary significantly depending on their diverse shapes and biological activities [48]. Glandular trichomes are considered key sites for the synthesis, secretion, and storage of secondary metabolites in plants [16]. In this study, we identified two representative forms of glandular trichomes in the leaves of *T. sinensis*: stalked glandular trichomes and sessile glandular trichomes (Figure 2). We found that these two types differ in cell number and morphology (Figure 2), indicating that their formation and development processes are regulated differently. Although the structure of the glandular trichomes of *T. sinensis* is similar to that of other plants, we currently lack sufficient understanding of the formation process of glandular trichomes, and the biosynthesis process of secondary metabolites in glandular trichomes is also poorly understood. In cannabis, both stalked and sessile glandular trichomes have been found. The stalked glandular trichomes are composed of 12 to 16 secretory disk cells, emit blue autofluorescence under the microscope, and are rich in cannabinoids and monoterpenes; in comparison, sessile glandular trichomes are composed of 8 secretory disk cells which contain fewer cannabinoids but a higher proportion of sesquiterpenes [28]. In tomatoes, various morphologies of glandular trichomes have been observed, including multiple-stalked cells, single-stalked cells, multiple-capitate cells, and single-capitate cells [49]. The type-VI glandular trichomes, composed of multiple stalked cells and capitate cells, serve as the primary sites for the production and storage of volatile monoterpenes and sesquiterpene compounds [50]. In general, regardless of the structural characteristics of glandular trichomes, most studies focus on the relationship between glandular trichome development and related secondary metabolites, especially within glandular trichomes (e.g., monoterpenes, sesquiterpenes, etc.) in biosynthesis, secretion, and storage [51]. We found that glandular trichomes in *T. sinensis* have many shapes, and the development process of sessile and stalked glandular trichomes needs further study. We still do not know whether there are differences in the types of metabolites produced by these two types of glandular trichomes, and whether the formation and development of different types of glandular trichomes are specifically regulated.

Despite the significant insights gained from this study, several challenges remain. First, the isolation and characterization of trichome-specific genes in *T*. *sinensis* were technically demanding due to the complexity of trichome structures and the low abundance of target transcripts. Second, the quantification of volatile compounds using GC-MS, while highly sensitive, required careful optimization to avoid artifacts and ensure reproducibility. These challenges highlight the need for more advanced techniques, such as single-cell RNA sequencing, to precisely capture trichome-specific gene expression profiles. Additionally, the integration of metabolomics and transcriptomics data could provide a more comprehensive understanding of the regulatory networks underlying volatile biosynthesis. Our work also opens new methodological perspectives for studying other woody vegetables or non-model plants. For instance, the identification of key terpene synthases in *T*. *sinensis* could serve as a foundation for developing metabolic engineering strategies to enhance the production of valuable sesquiterpenes in other crops. These advancements would not only address the current limitations but also pave the way for innovative approaches in plant biology and agricultural biotechnology.

## 4. Materials and Methods

### 4.1. Plant Materials

The *T. sinensis* varieties CAF0845 and CAF0830 used in this study were preserved in the *T. sinensis* germplasm resource nursery at the Institute of Subtropical Forestry of the Chinese Academy of Forestry in Hangzhou, Zhejiang Province [52]. The CAF0845 variety originated from Chongqing Province, while the CAF0830 variety was collected from Henan Province. Arabidopsis thaliana and tobacco were cultured in a greenhouse at 25 °C, with 16 h of light and 8 h of darkness.

### 4.2. Detection of Volatile Substances

In this study, the leaves were analyzed for volatile compounds using an Agilent 8890-5977B instrument (Agilent Technologies, La Jolla, CA, USA), following a headspace-solid phase microextraction gas chromatography–mass spectrometry (HS-SPME/GC-MS) protocol for scanning electron microscopy experiments. The specific treatments are outlined as follows: First, 0.1 to 0.2 g (accurate to 0.0001 g) of the ground plant sample was weighed into a 20 mL headspace vial, and then 50 μL of ethyl decanoate solution (10 μg/mL) was added as an internal standard. We secured the headspace vial with a threaded cap and *T. sinensis* with a silica gel spacer. We equilibrated the sample at 50 °C for 1 min in the CTC system. Subsequently, we extracted and adsorbed the sample by shaking it for 50 min. Finally, we desorbed the samples in the GC-MS inlet at 250 °C for 5 min [44].

The quantitative analysis of volatile compounds was performed using semi-quantitative estimation based on the internal standard with a correction factor. Decanoic acid ethyl ester (802180, Merck, Darmstadt, Germany) was added to the samples as the internal standard for calculation. Calibration curves were constructed for each compound using a series of diluted standards (concentration range: 0.1–100 µg/mL) with a linear regression coefficient (R^2^) ≥ 0.9. All the samples were analyzed in triplicate, and the final concentrations were reported as means with standard deviation (SD).

### 4.3. Scanning Electron Microscopic Observation of Trichomes and Statistical Analysis

The young leaves of *T. sinensis* were cut into 0.5 cm^2^ pieces and immediately placed in an FAA fixative solution. They were subjected to a vacuum for 15 min and then stored at 4 °C for approximately 24 h [53]. The samples were dehydrated using a CPD-300 critical point dryer (EM CPD300, Leica Microsystems, Wetzlar, Germany) and observed with a scanning electron microscope (Hitachi S-3400N, Hitachi, Tokyo, Japan). Photographs of the leaves from the same area (square millimeters) were selected, and the number of epidermal trichomes within the selected area was counted.

### 4.4. RNA Library Construction and Transcriptome Analysis

Total RNA was used as the input material for the RNA sample preparations. Sequencing libraries were generated using the NEBNext Ultra RNA Library Prep Kit for Illumina (NEB, Ipswich, MA, USA), and index codes were added to associate sequences with each sample. The original fluorescence image files obtained from the Illumina platform were transformed into short reads (raw data) through base calling, and these short reads were recorded in the FASTQ format [54], which contains both sequence information and corresponding sequencing quality metrics. Differentially expressed genes (DEGs) between CAF0845 and CAF0830 were identified using the DESeq2 program (https://www.omicshare.com/tools/, accessed on 4 November 2024). Subsequently, we performed Gene Ontology (GO) and Kyoto Encyclopedia of Genes and Genomes (KEGG) enrichment analyses.

### 4.5. RNA Extraction and Quantitative Real-Time PCR

Total RNA was extracted using a Total RNA Rapid Extraction Kit (Magen, Guangzhou, China), and first-strand cDNA synthesis was conducted with the PrimeScript™ RT Reagent Kit with gDNA Eraser (Takara, Dalian, China). Quantitative real-time PCR (qRT-PCR) was conducted using TB Green^®^ Ex Taq™ II (Takara, Dalian, China) on the QuantStudio™ 7 Flex (Applied Biosystems^®^ QuantStudio™ 7 Flex, Thermo Fisher, Waltham, MA, USA) with the following amplification program: one cycle of 30 s at 95 °C, followed by 40 cycles of 5 s at 95 °C and 30 s at 60 °C. The primer sequences used for qRT-PCR are listed in Supplementary Materials Table S5.

### 4.6. Isolation and Characterization of TsTPS1262 and TsTPS466

The candidate genes *TsTPS1262* and *TsTPS466* were isolated through polymerase chain reaction (PCR) amplification, with the *T. sinensis* cDNA serving as the template and the primers listed in Table S5. The PCR products were separated using gel electrophoresis, and the target fragments were purified and cloned with the T-Vector pMD™20 (Takara, Dalian, China). We utilized BioEdit to conduct multiple sequence comparisons of two cloned *T. sinensis* TPS genes alongside the TPS genes from various species, including Arabidopsis and rice. We conducted a phylogenetic tree analysis of the Arabidopsis TPS genes and the two cloned *T. sinensis* TPS genes using MEGA11 (V11.0.13). The bootstrap consensus tree was exported in the Newick file format and modified using the iTOL v7 online tool (https://itol.embl.de/, accessed on 23 October 2024).

### 4.7. Arabidopsis Transformation and Subcellular Localization

Constructs for the overexpression of *TsTPS1262* and *TsTPS466* were made by using the pNC vector system through the Nimble Cloning system [55]. We transformed *Arabidopsis* using the flower dip method [56] and screened T2 generation transgenic *Arabidopsis* seeds for one week on an MS medium containing hygromycin. The screened plants that exhibited hygromycin resistance were subsequently transplanted into the soil. We utilized *Arabidopsis* overexpression lines to investigate the subcellular localization of the *TsTPS1262* gene. We observed the roots and leaves of overexpressed *Arabidopsis* plants using a laser confocal microscope (LSM900, Zeiss, Oberkochen, Germany). We detected GFP signals with excitation in the 480–507 nm range and chloroplast autofluorescence with excitation in the 670–690 nm range.

### 4.8. Ectopic Expression of Target Gene in T. sinensis and Tobacco

*Agrobacterium tumefaciens* GV3101psoup (Weidi, Shanghai, China), which carries the overexpression plasmids of *TsTPS1262* and *TsTPS466*, were overexpressed in *T. sinensis*. The RUBY-reporter was obtained from Addgene (Plasmid #160906). The experimental steps were as follows: First, *T. sinensis* sterile seedlings from tissue culturing, approximately 2 weeks old, were completely submerged in an agrobacterial solution and were then subjected to vacuum treatment for 30 min. Finally, the surface of *T. sinensis* seedlings was air-dried for about 20 min, and they were then reintroduced into the MS medium for co-culture for 2 to 3 days to check the gene expression. The infiltration of tobacco leaves was carried out based on the previous report [57].

## Figures and Tables

**Table 1:** The GC-MS analysis of the volatiles of the varieties of *Toona sinensis*. Only the substance with more than 1% of the total is listed.

CAF0646 Category-I					
RT (min)	Compound Name	Matching Factor	Molecular Formula	CAS Number	Compound Content µg/g (Mean ± SD)
31.45	Copaene	97.89	C15H24	3856-25-5	44.67 ± 6.56
25.36	2-Mercapto-3,4-dimethyl-2,3-dihydrothiophene	77.90	C6H10S2	1000322-30-1	27.68 ± 4.07
30.52	alpha.-Cubebene	97.34	C15H24	17699-14-8	17.68 ± 3.59
14.44	alpha.-Pinene	98.37	C10H16	80-56-8	15.54 ± 2.51
34.75	2-Isopropenyl-4a,8-dimethyl-octahydronaphthalene	94.88	C15H24	207297-57-2	13.95 ± 2.62
30.16	Cyclohexene	96.52	C15H24	20307-84-0	13.88 ± 4.16
32.71	Caryophyllene	98.92	C15H24	87-44-5	12.61 ± 2.83
37.13	Disulfide, 1-(1-propenylthio)propyl propyl	96.41	C9H18S3	143193-11-7	12.48 ± 1.50
46.11	7-chromen-2-one acetate	72.25	C26H32O5	1000495-08-0	12.39 ± 2.44
34.59	Naphthalene	96.14	C15H24	17066-67-0	12.27 ± 2.73
22.88	1-((E)-Prop-1-en-1-yl)-2-((Z)-prop-1-en-1-yl)disulfane	97.71	C6H10S2	121609-82-3	11.01 ± 1.36
34.33	8-Isopropyl-1-methyl-3-methylenetricyclo-decane	98.51	C15H24	18252-44-3	7.15 ± 2.70
33.68	1,4,7,-Cycloundecatriene	98.74	C15H24	1000062-61-9	7.06 ± 1.45
31.53	Cyclohexane	96.98	C15H24	515-13-9	6.98 ± 1.84
33.19	7-Isopropyl-1,4-dimethyl-hexahydroazulene	98.04	C15H24	36577-33-0	4.91 ± 0.98
**CAF0830 Category-V**					
**RT (min)**	**Compound Name**	**Matching Factor**	**Molecular Formula**	**CAS Number**	**Compound Content µg/g (Mean ± SD)**
32.80	Caryophyllene	97.89	C15H24	87-44-5	84.82 ± 25.97
33.69	1,4,7,-Cycloundecatriene	99.12	C15H24	1000062-61-9	19.48 ± 4.97
34.62	alpha.-Farnesene	97.56	C15H24	502-61-4	11.57 ± 0.83
23.59	2-Mercapto-3,4-dimethyl-2,3-dihydrothiophene	84.13	C6H10S2	1000322-30-1	9.69 ± 5.11
31.73	Cyclohexane	97.10	C15H24	515-13-9	9.14 ± 4.49
31.40	Copaene	98.44	C15H24	3856-25-5	7.72 ± 3.97
32.26	Bicyclo[7.2.0]undec-4-ene, 4,11,11-trimethyl-8-methylene-	98.94	C15H24	13877-93-5	6.99 ± 4.24
22.87	1-((E)-Prop-1-en-1-yl)-2-((Z)-prop-1-en-1-yl)disulfane	97.85	C6H10S2	121609-82-3	3.71 ± 1.76
12.93	Thiophene, 2,4-dimethyl-	97.43	C6H8S	638-00-6	3.12 ± 1.61
23.03	1,2-Di((Z)-prop-1-en-1-yl)disulfane	92.64	C6H10S2	23838-22-4	2.64 ± 1.23
46.33	Aristol-1(10)-en-9-yl isovalerate	75.88	C20H32O2	1000414-30-6	2.46 ± 1.13
34.32	8-Isopropyl-1-methyl-3-methylenetricyclo-decane	98.57	C15H24	18252-44-3	2.17 ± 0.92
46.09	7-chromen-2-one acetate	73.40	C26H32O5	1000495-08-0	2.29 ± 1.27
34.22	alpha.-Bergamotene	97.72	C15H24	18252-46-5	1.73 ± 0.10
37.13	Disulfide, 1-(1-propenylthio)propyl propyl	91.14	C9H18S3	143193-11-7	1.70 ± 0.18
34.70	Cyclohexene	80.25	C15H24	20307-84-0	2.43 ± 0.92
39.98	Tripropyl orthoformate	90.55	C10H22O3	621-76-1	2.71 ± 2.33

## Data Availability

All data are provided and associated with the manuscript and can be obtained from the website of the manuscript. The raw sequencing dataset of the transcriptomes of *Toona sinensis* is stored in the National Genomics Data Center under bioproject No. PRJCA035282.

## Supplementary Materials

The following supporting information can be downloaded at https://www.mdpi.com/article/10.3390/ijms26041578/s1.

## References

[1] Peng W., Liu Y., Hu M., Zhang M., Yang J., Liang F., Huang Q., Wu C. (2018). Toona sinensis: A comprehensive review on its traditional usages, phytochemisty, pharmacology and toxicology. Rev. Bras. Farmacogn..

[2] Sun X., Zhang L. (2016). Quantitative Analysis and Comparison of Four Major Flavonol Glycosides in the Leaves of *Toona sinensis* (A. Juss.) Roemer (Chinese Toon) from Various Origins by High-Performance Liquid Chromatography-Diode Array Detector and Hierarchical Clustering Analysis. Pharmacogn. Mag..

[3] Liu C., Zhang J., Zhou Z., Hua Z., Wan H., Xie Y., Wang Z., Deng L. (2013). Analysis of Volatile Compounds and Identification of Characteristic Aroma Components of *Toona sinensis* (A. Juss.) Roem. Using GC-MS and GC-O. Food Nutr. Sci..

[4] Yang Y., Ma Y. (2020). Chemical components analysis of *Toona sinensis* bark and wood by pyrolisis–gas chromatography–mass spectrometry. Asia-Pac. J. Chem. Eng..

[5] Fan S., Chen H. (2007). *Toona sinensis* Roem (Meliaceae) leaf extract alleviates liver fibrosis via reducing TGFbeta1 and collagen. Food Chem. Toxicol..

[6] Liao N., Hu Z., Miao J., Hu X., Lyu X., Fang H., Zhou Y.-M., Mahmoud A., Deng G., Meng Y.-Q. (2022). Chromosome-level genome assembly of bunching onion illuminates genome evolution and flavor formation in Allium crops. Nat. Commun..

[7] Marcinkowska M.A., Jeleń H.H. (2022). Role of Sulfur Compounds in Vegetable and Mushroom Aroma. Molecules.

[8] Zhang L., Zhang Y. (2022). Differences in Volatile Organic Compounds of *Toona sinensis* from Eight Production Regions Analyzed by Gas Chromatography-Ion Mobility Spectrometry Combined with Chemometrics. Food Sci..

[9] Liu C., Zhang J. (2013). Comparative Analysis of Volatile Components in Three Cultivars of Chinese Toon (*Toona sinensis*) by GC-MS. Food Sci..

[10] Perestrelo R., Silva C., Câmara J.S. (2019). Off-Flavors in Alcoholic Beverages: An Overview. Food Aroma Evolution.

[11] Ibrahim M.R.S., Abdallah M.H. (2016). Naturally occurring thiophenes: Isolation, purification, structural elucidation, and evalua-tion of bioactivities. Phytochem. Rev..

[12] Margl L., Eisenreich W., Adam P., Bacher A., Zenk M.H. (2001). Biosynthesis of thiophenes in *Tagetes patula*. Phytochemistry.

[13] Wang C., Fu C., Li Y., Zhang Y., Zhang B., Zhang J. (2022). Integrated volatilomic profiles and chemometrics provide new insights into the spatial distribution and aroma differences of volatile compounds in seven *Toona sinensis* cultivars. Food Chem..

[14] Chen F., Tholl D., D’Auria J.C., Farooq A., Pichersky E., Gershenzon J. (2003). Biosynthesis and Emission of Terpenoid Volatiles from Arabidopsis Flowers. Plant Cell.

[15] Huchelmann A., Gastaldo C., Veinante M. (2014). S-carvone suppresses cellulase-induced capsidiol production in *Nicotiana taba-cum* by interfering with protein isoprenylation. Plant Physiol..

[16] Huchelmann A., Boutry M., Hachez C. (2017). Plant Glandular Trichomes: Natural Cell Factories of High Biotechnological Interest. Plant Physiol..

[17] Dai J., Wang M., Yin H., Han X., Fan Y., Wei Y., Lin J., Liu J. (2024). Integrating GC-MS and comparative transcriptome analysis reveals that TsERF66 promotes the biosynthesis of caryophyllene in *Toona sinensis* tender leaves. Front. Plant Sci..

[18] Zhang B., Hao L. (2024). Integration of transcriptome, volatile and non-volatile metabolite profile reveals characteristic aroma for-mation in Toona sinensis. Food Chem..

[19] Zhang L., Wei Y. (2024). Analysis of the Effect of Drying Methods on Volatile Components of *Toona sinensis* Based on GC-IMS and GC-MS Combined with Chemometrics. Sci. Technol. Food Ind..

[20] Yang W., Cadwallader K.R., Liu Y., Huang M., Sun B. (2019). Characterization of typical potent odorants in raw and cooked *Toona sinensis* (A. Juss.) M. Roem. by instrumental-sensory analysis techniques. Food Chem..

[21] Sun X., Yu P. (2019). Analysis of Volatile Components in Vacuum Freeze-dried *Toona sinensis* by HS-SPME Combined with GC-MS. Sci. Technol. Food Ind..

[22] Zhai X., Granvogl M. (2019). Characterization of the key aroma compounds in two differently dried *Toona sinensis* (A. Juss.) Roem. by means of the molecular sensory science concept. J. Agric. Food Chem..

[23] Tholl D., Chen F. (2005). Two sesquiterpene synthases are responsible for the complex mixture of sesquiterpenes emitted from Ara-bidopsis flowers: Sesquiterpene synthases in Arabidopsis flower. Plant J..

[24] Bohlmann J., Phillips M. (1999). cDNA cloning, characterization, and functional expression of four new monoterpene synthase members of the TPS gene family from grand fir (Abies grandis). Arch Biochem. Biophys.

[25] Frank L., Wenig M., Ghirardo A., van der Krol A., Vlot A.C., Schnitzler J., Rosenkranz M. (2021). Isoprene and β-caryophyllene confer plant resistance via different plant internal signalling pathways. Plant, Cell Environ..

[26] Huang M., Sanchez-Moreiras A.M. (2012). The major volatile organic compound emitted from Arabidopsis thaliana flowers, the sesquiterpene (E)-β-caryophyllene, is a defense against a bacterial pathogen. New Phytol..

[27] Mischko W., Hirte M., Fuchs M., Mehlmer N., Brück T.B. (2018). Identification of sesquiterpene synthases from the Basidiomycota *Coniophora puteana* for the efficient and highly selective β-copaene and cubebol production in E. coli. Microb. Cell Factories.

[28] Livingston S.J., Quilichini T.D. (2020). Cannabis glandular trichomes alter morphology and metabolite content during flower mat-uration. Plant J..

[29] Wagner G.J., Wang E., Shepherd R.W. (2004). New Approaches for Studying and Exploiting an Old Protuberance, the Plant Trichome. Ann. Bot..

[30] Schilmiller A.L., Last R.L., Pichersky E. (2008). Harnessing plant trichome biochemistry for the production of useful compounds. Plant J..

[31] Tissier A. (2012). Glandular trichomes: What comes after expressed sequence tags?. Plant J..

[32] Schuurink R., Tissier A. (2020). Glandular trichomes: Micro-organs with model status?. New Phytol..

[33] Glas J.J., Schimmel B.C.J., Alba J.M., Escobar-Bravo R., Schuurink R.C., Kant M.R. (2012). Plant Glandular Trichomes as Targets for Breeding or Engineering of Resistance to Herbivores. Int. J. Mol. Sci..

[34] Schilmiller A.L., Schauvinhold I. (2009). Monoterpenes in the glandular trichomes of tomato are synthesized from a neryl diphos-phate precursor rather than geranyl diphosphate. Proc. Natl. Acad. Sci. USA.

[35] Chen F., Tholl D., Bohlmann J., Pichersky E. (2011). The family of terpene synthases in plants: A mid-size family of genes for specialized metabolism that is highly diversified throughout the kingdom. Plant J..

[36] Falara V., Akhtar T.A., Nguyen T.T., Spyropoulou E.A., Bleeker P.M., Schauvinhold I., Matsuba Y., Bonini M.E., Schilmiller A.L., Last R.L. (2011). The Tomato Terpene Synthase Gene Family. Plant Physiol..

[37] Liu W., Lv H. (2017). New advances in the study of plant terpene synthases. Plant Physiol. J..

[38] Kabir N., Wang X., Lu L., Qanmber G., Liu L., Si A., Zhang L., Cao W., Yang Z., Yu Y. (2023). Functional characterization of TBL genes revealed the role of GhTBL7 and GhTBL58 in cotton fiber elongation. Int. J. Biol. Macromol..

[39] Zhou F., Pichersky E. (2020). The complete functional characterisation of the terpene synthase family in tomato. New Phytol..

[40] Wang Q. (2025). Expansion and functional divergence of terpene synthase genes in angiosperms: A driving force of terpene diversity. Hortic. Res..

[41] Zhao M., Li H. (2024). Traditional Uses, Chemical Constituents and Pharmacological Activities of the *Toona sinensis* Plant. Molecules.

[42] Wang C., Zhang B., Li Y., Hou J., Fu C., Wang Z., Zhang J. (2023). Integrated transcriptomic and volatilomic profiles to explore the potential mechanism of aroma formation in Toona sinensis. Food Res. Int..

[43] Dueholm B., Drew D.P., Sweetman C., Simonsen H.T. (2018). In planta and in silico characterization of five sesquiterpene synthases from Vitis vinifera (cv. Shiraz) berries. Planta.

[44] Hsu C.-Y., Huang P.-L., Chen C.-M., Mao C.-T., Chaw S.-M. (2012). Tangy scent in *Toona sinensis* (Meliaceae) leaflets: Isolation, functional characterization, and regulation of TsTPS1 and TsTPS2, two key terpene synthase genes in the biosynthesis of the scent compound. Curr. Pharm. Biotechnol..

[45] Liu X., Hao N., Feng R., Meng Z., Li Y., Zhao Z. (2021). Transcriptome and metabolite profiling analyses provide insight into volatile compounds of the apple cultivar ‘Ruixue’ and its parents during fruit development. BMC Plant Biol..

[46] Demurtas O.C., Nicolia A., Diretto G. (2023). Terpenoid Transport in Plants: How Far from the Final Picture?. Plants.

[47] Yang Q., Liu S. (2022). Cloning and Functional Characterization of Sesquiterpene Synthase Gene NtTPS21 in Tobacco. Chin. Tob. Sci..

[48] Hülskamp M. (2019). Trichomes. Curr. Biol..

[49] Simmons A.T., Gurr G.M. (2006). Trichomes of *Lycopersicon* species and their hybrids: Effects on pests and natural enemies. Agric. For. Entomol..

[50] Yang C., Marillonnet S., Tissier A. (2021). The scarecrow-like transcription factor SlSCL3 regulates volatile terpene biosynthesis and glandular trichome size in tomato (*Solanum lycopersicum*). Plant J..

[51] Turner G.W., Gershenzon J., Croteau R.B. (2000). Development of Peltate Glandular Trichomes of Peppermint. Plant Physiol..

[52] Dai J., Fan Y., Diao S., Yin H., Han X., Liu J. (2023). Construction of Core Collection and Phenotypic Evaluation of *Toona sinensis*. Forests.

[53] Chen C., Liu M. (2014). Transcriptome profiling reveals roles of meristem regulators and polarity genes during fruit trichome de-velopment in cucumber. J. Exp. Bot..

[54] Cock P.J.A., Fields C.J., Goto N., Heuer M.L., Rice P.M. (2009). The Sanger FASTQ file format for sequences with quality scores, and the Solexa/Illumina FASTQ variants. Nucleic Acids Res..

[55] Yan P., Tuo D., Shen W., Deng H., Zhou P., Gao X. (2022). A Nimble Cloning-compatible vector system for high-throughput gene functional analysis in plants. Plant Commun..

[56] Zhang X., Henriques R. (2006). Agrobacterium-mediated transformation of Arabidopsis thaliana using the floral dip method. Nat. Protoc..

[57] Zhang Y., Li Y. (2022). Effects of three kinds of Agrobacterium and different transformation conditions on the transient expression of GFP in *Nicotiana benthamiana*. Bull. Bot..

