# Supplementary material for: Two Terpene Synthases Are Involved in Multiple Sesquiterpene Biosynthesis in the Woody Vegetable, Toona sinensis
Source: Int J Mol Sci. 2025 Feb 13;26(4):1578. doi: 10.3390/ijms26041578 (PMC11855491; doi:10.3390/ijms26041578)
Supplement: Supplementary file 1 [file ijms-26-01578-s001.zip › Supplementary FigureS1-2.pdf]

Figure S1. Heatmap of gene expression correlation between transcriptome sequencing samples.

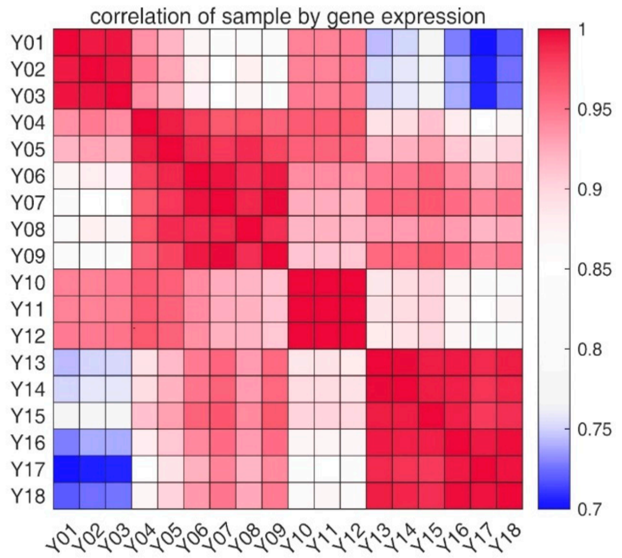

Figure S1 A heatmap illustrating the correlation of transcriptome gene expression among the samples of different leaf zones in the *T. sinensis* varieties with varying trichome densities.

Figure S2. The expression of all TsTPS gene expressions in transcriptome data.

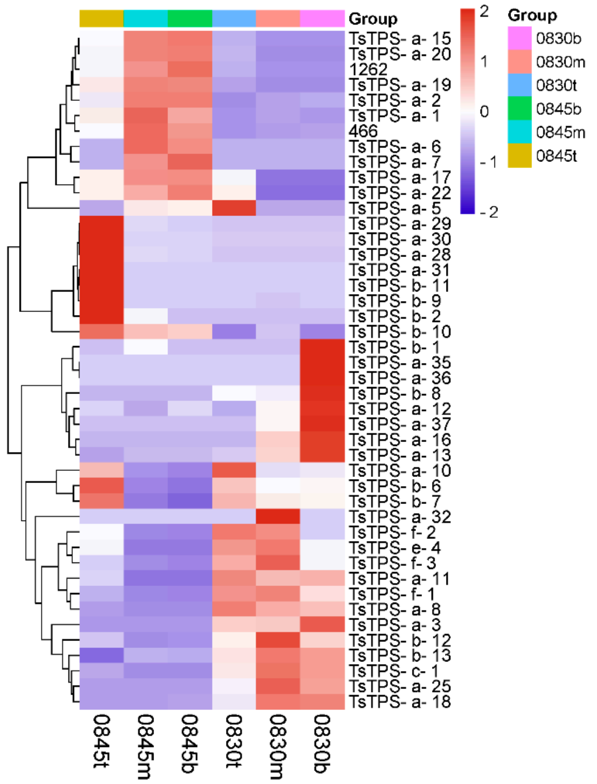

Figure S2 A heatmap illustrating the transcriptome expression of all the TPS genes among the samples of different leaf zones in the *T. sinensis* varieties with varying

trichome densities.
